# Correlation of the triglyceride-glucose index with major adverse cardiovascular events in type 2 diabetes mellitus patients with acute myocardial infarction combined with HFpEF

**DOI:** 10.3389/fendo.2025.1585067

**Published:** 2025-08-26

**Authors:** Xiaodong Zhang, Nan Niu, Shengqin Yu, Xinxin Zhang, Xuefu Chen, Ming Yu, Wenmiao Zhang, Ying Liu, Zhenwei Wang

**Affiliations:** ^1^ Department of Cardiology, The First Affiliated Hospital of Dalian Medical University, Dalian, China; ^2^ Department of Cardiology, The Second Affiliated Hospital of Dalian Medical University, Dalian, China; ^3^ Department of Cardiology, The First Affiliated Hospital of Zhengzhou University, Zhengzhou, China

**Keywords:** triglyceride-glucose index, acute myocardial infarction, heart failure with preserved ejection fraction, major adverse cardiovascular events, type 2 diabetes mellitus

## Abstract

**Aims:**

This study was conducted to evaluate the correlation between triglyceride-glucose index (TyG) and major adverse cardiovascular events (MACE) in patients with type 2 diabetes mellitus (T2DM) and heart failure with preserved ejection fraction (HFpEF) after acute myocardial infarction (AMI).

**Methods:**

This retrospective study at the First Affiliated Hospital of Dalian Medical University included 400 AMI patients with T2DM and HFpEF who underwent percutaneous coronary intervention (PCI) between 1 January 2018 and 1 January 2023. The study was conducted using univariate and multivariate Cox regression analyses, subgroup analyses, receiver operating characteristic (ROC) curves, and Kaplan–Meier survival curves to assess the correlation between the TyG index and MACE.

**Results:**

Multivariate Cox regression analyses showed that in model 3 with variables fully adjusted, when TyG was used as a categorical variable, the risk of MACE in the TyG T2 and T3 groups was 1.622 times and 2.247 times higher than that in the T1 group, respectively (*P* < 0.05). When TyG was used as a continuous variable, the risk of MACE increased by 49.5% for every 1 unit increase in the TyG index (*P* < 0.001). In the subgroup analysis, elevated TyG index levels were consistently associated with an increased risk of MACE across multiple clinical subgroups (*P* < 0.05). ROC analysis showed that the TyG index significantly predicted the occurrence of MACE (AUC: 0.635, 95% CI: 0.580–0.691, *P* < 0.001), all-cause death (AUC: 0.565, 95% CI: 0.508–0.622, *P* = 0.027), non-fatal myocardial infarction (AUC: 0.617, 95% CI: 0.542–0.693, *P* = 0.004), and unplanned revascularization (AUC: 0.644, 95% CI: 0.578–0.710, *P* < 0.001). The Kaplan–Meier survival curves revealed statistically significant differences in survival probabilities for the occurrence of MACE, all-cause death, non-fatal myocardial infarction, and unplanned revascularization across the three TyG index groups as the follow-up period progressed (*P* < 0.05).

**Conclusions:**

The TyG index was independently associated with MACE in T2DM patients with AMI combined with HFpEF.

## 1 Introduction

Over the past three decades, significant advances have been made in the treatment of coronary heart disease (CHD) and acute myocardial infarction (AMI). However, AMI, the most lethal and prevalent form of CHD, continues to be the most serious and dangerous type, remaining the leading cause of heart failure (HF) (1, 2). According to a systematic review and meta-analysis published in 2023 (with data updated through September 2022), the global prevalence of MI is 3.8% in individuals under 60 years old and rises to 9.5% in those over 60, indicating a marked age-related increase (3). MI is not only a critical manifestation of CHD but also a major precipitating factor for HF. In recent years, there has been increased attention on MI-related HF, particularly in the context of metabolic dysfunction. The prognostic value of this condition in CHD patients with type 2 diabetes mellitus (T2DM) is of paramount importance. Given that diabetes accelerates atherosclerosis and increases the risk of both MI and subsequent HF, understanding the interplay between these conditions is essential for improving risk stratification and guiding targeted interventions.

Several factors contribute to the risk of AMI, including poor glycemic control, hypertension, hyperlipidemia, mental stress, air pollution, and obesity (4, 5). If these risk factors are not effectively managed, they can lead to adverse left ventricular remodeling, thereby exacerbating the incidence of HF following AMI (6). Moreover, the prognosis of HF after AMI is notably worse in patients with T2DM compared to those without glucose disorders (7, 8). Furthermore, in a large cohort of 4,082 Chinese patients with HF, the 12-month follow-up revealed a high all-cause mortality rate of 19.6%, a rehospitalization rate of 24.4%, and a composite event rate of 40.15%, with overall health-related quality of life (HRQL) being poor as indicated by a mean MLHFQ score of 42.9—significantly higher in women than in men—and HRQL independently predicting both all-cause mortality and HF hospitalization (9). Despite this, current research predominantly focuses on the prevention and treatment of ischemic HF, with little attention given to further classifying HF post-AMI or exploring the link between glycemic metabolism abnormalities and HF onset, particularly in the context of heart failure with preserved ejection fraction (HFpEF) (10). This research gap is of critical importance, as HFpEF now accounts for approximately half of all HF cases and is closely associated with metabolic comorbidities such as diabetes, obesity, and hypertension, with emerging evidence indicating that systemic inflammation, microvascular endothelial dysfunction, and impaired myocardial energetics—often driven by glycemic dysregulation—play central roles in its pathogenesis (11). Therefore, it is crucial to examine whether risk factors associated with AMI in T2DM patients influence the outcomes of HFpEF or affect long-term cardiovascular outcomes following AMI.

One key factor in the development of cardiovascular diseases (CVD) is insulin resistance (IR), which is often a hallmark of metabolic disorders and systemic inflammation (12). IR frequently coexists with obesity, hypertension, and dyslipidemia, all of which are significant risk factors for CVD development and prognosis. The triglyceride-glucose index (TyG), derived from fasting triglyceride (TG) and fasting plasma glucose (FPG) levels, has emerged as a reliable indicator of IR in high-risk populations (13). In addition to its association with diabetes, the TyG index is also strongly linked to hypertension, dyslipidemia, metabolic syndrome, cardiovascular diseases, and mortality (13–17). Furthermore, Sun et al. demonstrated in a retrospective study of 2,055 ischemic HF patients undergoing percutaneous coronary intervention (PCI) that the TyG index was independently and positively associated with the risk of major adverse cardiovascular events (MACE), with higher TyG levels corresponding to an increased incidence of adverse outcomes (18). Additionally, in a multicenter cohort study of 277 patients with newly diagnosed ischemic cardiomyopathy and HFpEF undergoing coronary artery bypass grafting (CABG), Ruan et al. demonstrated that the TyG index was an independent predictor of MACE, showing a linear positive association with risk, and that incorporating the TyG index into traditional cardiovascular risk models significantly improved prognostic accuracy through enhanced discrimination, calibration, and reclassification metrics (19).

However, despite the accumulation of substantial research evidence, some studies—particularly those focusing on patients with T2DM complicated by AMI and HFpEF—have yet to establish a clear association between the TyG index and MACE. This indicates that further validation is needed to confirm the predictive value of the TyG index for MACE in this specific patient population. Therefore, to address this research gap, the present study aims to focus on T2DM patients with AMI and HFpEF who have undergone interventional therapy, exploring the association between the TyG index and MACE.

## 2 Methods

### 2.1 Study population and grouping

This was a single-center, retrospective cohort study that included patients with T2DM and AMI who were admitted to the Department of Cardiology at the First Affiliated Hospital of Dalian Medical University for PCI. These patients were diagnosed with HFpEF between 1 January 2018 and 1 January 2023. Patients with end-stage hepatic or renal failure, coagulation abnormalities, aortic coarctation, or incomplete data, as well as those lost to follow-up or who did not undergo PCI, were excluded from the study. After excluding these individuals, a total of 400 patients were finally included in the analysis. All procedures were conducted in compliance with the Declaration of Helsinki and its amendments. The study protocol was approved by the Institutional Review Board of the First Affiliated Hospital of Dalian Medical University. Informed consent was obtained from all participants prior to the collection of clinical data.

### 2.2 Data collection and definitions

All clinical data and study information were collected from Yidu Cloud, one of the largest medical databases in China, at the First Hospital of Dalian Medical University. These data included patient demographics, comorbidities, medication information, anthropometrics, blood biomarkers, medication regimens, echocardiographic results, and data related to PCI procedures.

Demographic data comprised age, gender, smoking, and family history of CHD. Smoking was defined as continuous or cumulative smoking for 6 months or more prior to enrollment. A CHD family history was defined as a genetic predisposition to the disease, with at least two or more close relatives affected.

Comorbidity data included hypertension, stroke, and atrial fibrillation (AF). Hypertension in adults was diagnosed based on a systolic blood pressure (SBP) ≥140 mmHg and/or a diastolic blood pressure (DBP) ≥90 mmHg (20). Diabetes was diagnosed in patients with symptoms such as polydipsia, polyuria, polyphagia, and weight loss, combined with a blood glucose level greater than 11.1 mmol/L at any time, a fasting blood glucose greater than 7.0 mmol/L, or hemoglobin A1c (HbA1c) ≥6.5%, or a 2-h oral glucose tolerance test blood glucose greater than 11.1 mmol/L (21). Stroke was defined as the impairment of blood circulation in the brain, leading to brain tissue damage due to the obstruction or rupture of cerebral blood vessels, including both ischemic and hemorrhagic stroke types (22). AF was defined as a rapid arrhythmia with disordered electrical activity in the atria, resulting in irregular and rapid fibrillation waves. The study included all forms of AF, including first diagnosis, paroxysmal, persistent, long-term persistent, and permanent atrial fibrillation (23). HFpEF was diagnosed based on the fulfillment of all the following three criteria: 1) the presence of typical HF symptoms and/or signs, such as shortness of breath, fatigue, or reduced exercise capacity; 2) a left ventricular ejection fraction (LVEF) of 50% or higher; and 3) objective indicators of diastolic dysfunction and/or elevated left ventricular filling pressures (24). These indicators included structural abnormalities (e.g., left atrial volume index >34 mL/m^2^, left ventricular mass index ≥95 g/m^2^ in women or ≥115 g/m^2^ in men, or relative wall thickness >0.42), functional impairments (e.g., E/e′ ratio >9, tricuspid regurgitation velocity >2.8 m/s, or pulmonary artery systolic pressure >35 mmHg), and elevated levels of natriuretic peptides [N-terminal pro-B-type natriuretic peptide (NT-proBNP) >125 pg/mL or B-type natriuretic peptide (BNP) >35 pg/mL in sinus rhythm; NT-proBNP >365 pg/mL or BNP >105 pg/mL in atrial fibrillation] (24).

Anthropometric data included body mass index (BMI), SBP, and DBP. BMI was calculated using the formula: BMI = weight (kg)/height (m)^2^. Additional data collected included the presence of ST-segment elevation myocardial infarction (STEMI) and Killip classification. STEMI was defined as marked ST-segment elevation on the electrocardiogram, usually caused by the rupture of an intracoronary plaque or thrombosis leading to coronary occlusion, which results in sustained myocardial ischemia and hypoxia, ultimately causing myocardial necrosis (25). The Killip classification, a grading system for assessing the cardiac functional status of patients with AMI, is divided into four grades (I–IV), with the condition progressively worsening (26).

Hematological biomarkers included FPG, HbA1c, albumin, uric acid (UA), estimate glomerular filtration rate (eGFR) [calculated using the Modification of Diet in Renal Disease (MDRD) equation: eGFR = 175 × (serum creatinine [(mg/dL)])^–1.234^ × (age [years])^–0.179^ × 0.79 (if female)] (27), TG, total cholesterol (TC), high-density lipoprotein cholesterol (HDL-C), low-density lipoprotein cholesterol (LDL-C), fibrinogen (FIB), D-dimer, high-sensitivity C-reactive protein (Hs-CRP), cardiac biomarkers (troponin I), and B-type natriuretic peptide (BNP).

Discharge medication data included the use of antiplatelet agents (such as aspirin, clopidogrel, and ticagrelor), statins, angiotensin-converting enzyme inhibitors (ACEIs), angiotensin II receptor blockers (ARBs), and β-blockers. Echocardiographic data included LVEF. All echocardiographic data were recorded by an experienced cardiac sonographer using a cardiac ultrasound machine. Procedure-related data included details on the multivessel disease. Multivessel disease was defined as lesions involving two or more coronary arteries with ≥50% stenosis.

### 2.3 Study endpoints and follow-up

In this study, patients were enrolled for follow-up starting from the date of their first hospitalization, with the follow-up period extending until either the patient’s death or 31 July 2024. The median follow-up time was 24.63 months. The study endpoint was MACE, defined as a composite of one or more of the following: all-cause death, unplanned revascularization, and non-fatal myocardial infarction. To identify clinical characteristics associated with adverse cardiovascular outcomes, baseline variables were compared between patients with and without MACE. This grouping approach was intended to explore potential risk factors for MACE. All enrolled patients were encouraged to monitor their condition regularly through outpatient services. For those who did not complete the follow-up program, efforts were made to contact them by telephone to ensure data completeness.

### 2.4 Calculation method of the TyG index

FPG and TG levels were collected for all patients during hospitalization. Specifically, blood samples were obtained in the early morning of the day following admission after an overnight fast of at least 8 h. All biochemical measurements were performed at the same clinical laboratory within the hospital using standardized procedures, ensuring consistency in both testing methods and fasting conditions. The formula for calculating the TyG index was as follows: TyG = Ln [fasting TG (mg/dL) × FPG (mg/dL)/2] (28). Based on the tertiles of the TyG index, patients were divided into three groups: T1 (≤8.76), T2 (8.77–9.51), and T3 (>9.51). This tertile-based stratification is widely used in metabolic and cardiovascular research to ensure statistical comparability across groups and avoid arbitrary threshold selection. Baseline characteristics were analyzed across these TyG tertiles to evaluate the association between metabolic risk status and clinical features or outcomes.

### 2.5 Statistical analysis

Statistical analyses were performed using SPSS statistical software version 26.0 (SPSS Inc., Chicago, IL, USA). Categorical variables were expressed as percentages. Continuous variables that were normally distributed were presented as means ± standard deviation, while non-normally distributed continuous variables were expressed as medians with interquartile ranges. To compare the differences between two or more groups, the chi-square test was used for categorical variables. For continuous variables, the independent samples *t*-test was applied for two-group comparisons with a normal distribution, while one-way ANOVA was used for comparisons involving three groups. For non-normally distributed data, the Mann–Whitney *U* test or Kruskal–Wallis test was applied, depending on the number of groups. Univariate and multivariate Cox regression analyses were performed to identify independent factors predicting the MACE. The proportional hazards assumption was tested using Schoenfeld residuals, and no significant violations were observed. Covariates included in the multivariate logistic regression analysis were those that showed a statistically significant association with MACE (*P* < 0.05) in the univariate logistic regression analysis. In addition, subgroup analyses were performed using Cox regression within different clinical subgroups (such as age, sex, hypertension, STEMI status, Killip classification, and multivessel disease) to evaluate the association between TyG tertiles and MACE in each category. The rationale for conducting subgroup analyses was to explore whether the predictive value of the TyG index for MACE remained consistent across various clinically relevant populations. These subgroups were selected based on their known associations with cardiovascular risk and their clinical importance in the context of HF, AMI, and MACE. Receiver operating characteristic (ROC) curve analysis was performed to evaluate the predictive power of the TyG index for the events. The area under the curve (AUC) was calculated for each endpoint to determine the diagnostic accuracy. Kaplan–Meier analysis was employed to estimate the cumulative incidence of clinical adverse events, while the log-rank test was applied to compare survival distributions across groups. A two-sided *P*-value of <0.05 was considered statistically significant.

## 3 Results

### 3.1 Baseline demographics and clinical characteristics

**Table 1** presents the clinical characteristics of the population, grouped according to the occurrence of MACE. The results showed that, compared to the group without MACE, the MACE group had a higher median age; a higher probability of being classified as Killip class III–IV; and elevated SBP, FPG, FIB, D-dimer, BNP, and TyG index levels. Moreover, the MACE group had higher rates of clopidogrel use and multivessel disease (*P* < 0.05). In contrast, the MACE group had lower rates of STEMI and use of aspirin and ticagrelor and lower levels of eGFR (*P* < 0.05).

**Table 1:** Clinical characteristics according to MACE.

Variables	Total population	Non-MACE	MACE	P-value
Age, years	74.39 ± 11.15	71.84 ± 10.93	75.88 ± 11.03	<0.001
Male, n (%)	209 (52.3)	84 (56.8)	125 (49.6)	0.167
Smoking, n (%)	121 (30.3)	48 (32.4)	73 (29.0)	0.466
STEMI, n (%)	172 (43.0)	82 (55.4)	90 (35.7)	<0.001
Killip class, n (%)				0.027
I	197 (49.3)	82 (55.4)	115 (45.6)	
II	147 (36.8)	55 (37.2)	92 (36.5)	
III	32 (8.0)	7 (4.7)	25 (9.9)	
IV	24 (6.0)	4 (2.7)	20 (7.9)	
Family history of CHD, n (%)	62 (15.5)	23 (15.5)	39 (15.5)	0.986
Hypertension, n (%)	278 (69.5)	95 (64.2)	183 (72.6)	0.077
Stroke, n (%)	53 (13.3)	18 (12.2)	35 (13.9)	0.623
AF, n (%)	36 (9.0)	8 (5.4)	28 (11.1)	0.054
BMI, kg/m2	26.76 ± 3.95	26.93 ± 4.27	26.66 ± 3.76	0.515
SBP, mmHg	132.11 ± 29.59	128.16 ± 28.67	134.43 ± 29.93	0.040
DBP, mmHg	74.74 ± 15.31	74.46 ± 15.69	74.90 ± 15.11	0.779
FPG, mmol/L	8.84 (5.80, 13.44)	6.36 (5.21, 8.84)	10.56 (6.61, 14.84)	<0.001
HbA1c, %	7.40 (6.13, 8.80)	7.30 (6.00, 8.90)	7.50 (6.30, 8.80)	0.163
TG, mmol/L	1.28 (0.94, 1.84)	1.37 (0.98, 1.82)	1.25 (0.92, 1.85)	0.465
TC, mmol/L	4.67 ± 1.29	4.73 ± 1.32	4.63 ± 1.27	0.423
LDL-C, mmol/L	2.92 ± 0.93	2.95 ± 0.88	2.90 ± 0.95	0.549
HDL-C, mmol/L	1.02 ± 0.29	1.04 ± 0.31	1.02 ± 0.28	0.443
Albumin, g/L	35.97 ± 3.96	36.21 ± 4.10	35.83 ± 3.87	0.354
UA, μmol/L	387.55 ± 127.87	394.12 ± 121.86	383.69 ± 131.35	0.432
eGFR, mL/min	67.50 (46.25, 85.00)	71.00 (53.25, 88.50)	65.00 (40.00, 84.75)	0.008
Hs-CRP, mg/L	44.70 (24.00, 88.80)	44.70 (21.75, 90.05)	44.80 (24.35, 87.50)	0.880
FIB, g/L	3.59 (2.78, 4.43)	3.35 (2.59, 4.15)	3.73 (2.93, 4.49)	0.029
D-dimer, mg/L	240.00 (0.77, 779.37)	130.00 (0.60, 630.00)	310.00 (1.14, 779.37)	0.004
Troponin I, ng/mL	8.16 (1.50, 48.94)	9.76 (1.96, 94.86)	7.63 (1.40, 39.11)	0.100
BNP, pg/mL	696.59 (523.73, 1,078.49)	650.83 (508.30, 958.26)	735.39 (529.34, 1,146.09)	0.015
Discharge medication, *n* (%)				
Aspirin	295 (73.8)	121 (81.8)	174 (69.0)	0.005
Clopidogrel	234 (58.5)	73 (49.3)	161 (63.9)	0.004
Ticagrelor	166 (41.5)	75 (50.7)	91 (36.1)	0.004
Statins	374 (93.5)	138 (93.2)	236 (93.7)	0.873
ACEI/ARB	308 (77.0)	106 (71.6)	202 (80.2)	0.050
β-Blockers	274 (68.5)	101 (68.2)	173 (68.7)	0.932
LVEF, %	54.73 ± 3.05	54.86 ± 2.91	54.66 ± 3.13	0.528
Multivessel disease, *n* (%)	189 (47.3)	59 (39.9)	130 (51.6)	0.023
TyG index	9.17 ± 0.75	8.95 ± 0.68	9.30 ± 0.77	<0.001

MACE, major adverse cardiovascular events; STEMI, ST-elevation myocardial infarction; CHD, coronary heart disease; AF, atrial fibrillation; BMI, body mass index; SBP, systolic blood pressure; DBP, diastolic blood pressure; FPG, fasting plasma glucose; HbA1c, hemoglobin A1c; TG, triglycerides; TC, total cholesterol; LDL-C, low-density lipoprotein cholesterol; HDL-C, high-density lipoprotein cholesterol; UA, uric acid; eGFR, estimated glomerular filtration rate; Hs-CRP, high-sensitivity C-reactive protein; FIB, fibrinogen; BNP, B-type natriuretic peptide; ACEI, angiotensin-converting enzyme inhibitor; ARB, angiotensin II receptor blocker; LVEF, left ventricular ejection fraction; TyG index, triglyceride-glucose index.

**Table 2** displays the clinical characteristics of the cohort, grouped according to TyG tertiles. The TyG tertiles were defined as follows: TyG-T1: ≤8.76, TyG-T2: 8.77–9.51, and TyG-T3: >9.51. The results indicated that significant differences were observed among the three TyG groups in terms of age, Killip classification, SBP, DBP, FPG, HbA1c, TG, TC, LDL-C, HDL-C, UA, eGFR, FIB, use of β-blockers, LVEF, all-cause death, MACE, non-fatal myocardial infarction, and unplanned revascularization (*P* < 0.05). Specifically, the incidence of all-cause death, MACE, non-fatal myocardial infarction, and unplanned revascularization increased with higher TyG tertile levels (*P* < 0.05).

**Table 2:** Clinical characteristics according to TyG tertiles.

Variables	T1	T2	T3	*P*-value
Age, years	76.70 ± 10.06	74.03 ± 11.16	72.44 ± 11.83	0.007
Male, *n* (%)	77 (57.9)	65 (48.9)	67 (50.0)	0.275
Smoking, *n* (%)	37 (27.8)	39 (29.3)	45 (33.6)	0.568
STEMI, *n* (%)	56 (42.1)	57 (42.9)	59 (44.0)	0.950
Killip class, *n* (%)				0.038
I	69 (51.9)	71 (53.4)	57 (42.5)	
II	50 (37.6)	49 (36.8)	48 (35.8)	
III	11 (8.3)	6 (4.5)	15 (11.2)	
IV	3 (2.3)	7 (5.3)	14 (10.4)	
Family history of CHD, *n* (%)	15 (11.3)	21 (15.8)	26 (19.4)	0.185
Hypertension, *n* (%)	85 (63.9)	91 (68.4)	102 (76.1)	0.091
Stroke, *n* (%)	20 (15.0)	14 (10.5)	19 (14.2)	0.515
AF, *n* (%)	13 (9.8)	6 (4.5)	17 (12.7)	0.061
BMI, kg/m^2^	26.50 ± 4.28	26.43 ± 3.75	27.36 ± 3.77	0.099
SBP, mmHg	129.37 ± 31.25	129.62 ± 24.89	137.30 ± 31.65	0.045
DBP, mmHg	72.95 ± 15.11	73.54 ± 12.84	77.71 ± 17.30	0.021
FPG, mmol/L	5.53 (4.85, 6.24)	9.02 (6.55, 12.35)	14.34 (10.99, 17.13)	<0.001
HbA1c, %	6.50 (5.80, 7.50)	7.50 (6.15, 8.75)	8.30 (7.28, 9.60)	<0.001
TG, mmol/L	0.92 (0.74, 1.18)	1.31 (1.01, 1.65)	2.04 (1.45, 2.87)	<0.001
TC, mmol/L	4.24 ± 1.19	4.57 ± 1.07	5.19 ± 1.41	<0.001
LDL-C, mmol/L	2.80 ± 0.84	2.88 ± 0.93	3.07 ± 1.00	0.048
HDL-C, mmol/L	1.06 ± 0.33	1.04 ± 0.27	0.96 ± 0.26	0.012
Albumin, g/L	35.70 ± 3.79	36.15 ± 3.87	36.07 ± 4.21	0.613
UA, μmol/L	388.10 ± 133.64	366.77 ± 122.97	407.63 ± 124.41	0.033
eGFR, mL/min	71.00 (50.50, 86.00)	70.00 (51.00, 93.50)	57.50 (36.50, 79.25)	0.002
Hs-CRP, mg/L	47.20 (24.00, 89.85)	48.20 (29.90, 92.10)	41.50 (21.30, 83.60)	0.130
FIB, g/L	3.32 (2.59, 4.04)	3.51 (2.71, 4.36)	3.73 (3.13, 4.99)	0.001
D-dimer, mg/L	290.00 (1.20, 779.37)	130.00 (0.63, 690.00)	215.00 (0.97, 779.37)	0.086
Troponin I, ng/mL	9.95 (1.33, 69.68)	6.41 (1.79, 52.51)	8.06 (1.46, 41.50)	0.934
BNP, pg/mL	697.62 (529.29, 1,067.11)	656.07 (501.76, 968.20)	755.61 (540.76, 1,137.00)	0.117
Discharge medication, *n* (%)				
Aspirin	89 (66.9)	104 (78.2)	102 (76.1)	0.084
Clopidogrel	88 (66.2)	69 (51.9)	77 (57.5)	0.058
Ticagrelor	45 (33.8)	64 (48.1)	57 (42.5)	0.058
Statins	126 (94.7)	127 (95.5)	121 (90.3)	0.177
ACEI/ARB	97 (72.9)	102 (76.7)	109 (81.3)	0.262
β-Blockers	81 (60.9)	87 (65.4)	106 (79.1)	0.004
LVEF, %	55.43 ± 3.05	54.40 ± 2.90	54.37 ± 3.09	0.005
Multivessel disease, *n* (%)	57 (42.9)	68 (51.1)	64 (47.8)	0.397
All-cause death, *n* (%)	52 (39.1)	43 (32.3)	66 (49.3)	0.018
MACE, *n* (%)	67 (50.4)	81 (60.9)	104 (77.6)	<0.001
Non-fatal myocardial infarction, *n* (%)	13 (9.8)	18 (13.5)	29 (21.6)	0.021
Unplanned revascularization, *n* (%)	10 (7.5)	28 (21.1)	34 (25.4)	<0.001

TyG, triglyceride-glucose index; STEMI, ST-elevation myocardial infarction; CHD, coronary heart disease; AF, atrial fibrillation; CKD, chronic kidney disease; BMI, body mass index; SBP, systolic blood pressure; DBP, diastolic blood pressure; FPG, fasting plasma glucose; HbA1c, hemoglobin A1c; TG, triglycerides; TC, total cholesterol; LDL-C, low-density lipoprotein cholesterol; HDL-C, high-density lipoprotein cholesterol; UA, uric acid; eGFR, estimated glomerular filtration rate; FIB, fibrinogen; BNP, B-type natriuretic peptide; ACEI, angiotensin-converting enzyme inhibitor; ARB, angiotensin II receptor blocker; LVEF, left ventricular ejection fraction; MACE, major adverse cardiovascular events.

### 3.2 Association between TyG and MACE

**Table 3** presents the results of the univariate Cox regression analysis for MACE. The analysis showed that age, STEMI, Killip classification III–IV, hypertension, SBP, FPG, eGFR, troponin I, BNP, aspirin, clopidogrel, ticagrelor, ACEI/ARB, multivessel disease, and the TyG index were all significantly correlated with the risk of MACE (*P* < 0.05).

**Table 3:** Univariate Cox regression analysis of MACE.

Variables	HR	95% CI	*P*-value
Age	1.026	1.013–1.039	<0.001
Male	0.834	0.651–1.068	0.151
Smoking	0.872	0.664–1.145	0.326
STEMI	0.599	0.463–0.776	<0.001
Killip class			
I	Ref		
II	1.186	0.902–1.561	0.222
III	1.605	1.041–2.476	0.032
IV	2.468	1.532–3.975	<0.001
Family history of CHD	1.092	0.776–1.536	0.615
Hypertension	1.382	1.048–1.824	0.022
Stroke	1.210	0.847–1.730	0.295
AF	1.472	0.993–2.182	0.054
BMI	0.995	0.965–1.025	0.720
SBP	1.006	1.002–1.010	0.004
DBP	1.001	0.993–1.009	0.757
FPG	1.068	1.048–1.089	<0.001
HbA1c	1.026	0.962–1.094	0.429
TG	1.026	0.904–1.165	0.689
TC	0.950	0.859–1.050	0.312
LDL-C	0.969	0.845–1.112	0.655
HDL-C	0.859	0.562–1.312	0.481
Albumin	0.986	0.956–1.017	0.378
UA	1.000	0.999–1.001	0.472
eGFR	0.993	0.989–0.997	0.001
Hs-CRP	1.000	0.998–1.002	0.890
FIB	1.049	0.963–1.143	0.272
D-dimer	1.000	1.000–1.000	0.321
Troponin I	0.999	0.998–1.000	0.047
BNP	1.000	1.000–1.000	0.001
Discharge medication			
Aspirin	0.634	0.485–0.829	0.001
Clopidogrel	1.402	1.084–1.814	0.010
Ticagrelor	0.713	0.551–0.922	0.010
Statins	1.085	0.654–1.800	0.752
ACEI/ARB	1.437	1.054–1.959	0.022
β Blockers	1.068	0.818–1.394	0.629
LVEF	0.987	0.949–1.027	0.529
Multivessel disease	1.318	1.029–1.688	0.029
TyG index	1.470	1.256–1.722	< 0.001

HR, hazard ratio; CI, confidence interval; MACE, major adverse cardiovascular events; STEMI, ST-elevation myocardial infarction; CHD, coronary heart disease; AF, atrial fibrillation; BMI, body mass index; SBP, systolic blood pressure; DBP, diastolic blood pressure; FPG, fasting plasma glucose; HbA1c, hemoglobin A1c; TG, triglycerides; TC, total cholesterol; LDL-C, low-density lipoprotein cholesterol; HDL-C, high-density lipoprotein cholesterol; UA, uric acid; eGFR, estimated glomerular filtration rate; Hs-CRP, high-sensitivity C-reactive protein; FIB, fibrinogen; BNP, B-type natriuretic peptide; ACEI, angiotensin-converting enzyme inhibitor; ARB, angiotensin II receptor blocker; LVEF, left ventricular ejection fraction; TyG, triglyceride-glucose index.

**Table 4** displays the results of the multivariate Cox regression analyses for TyG and MACE. In the unadjusted model 1, as well as in model 2 (which was adjusted for age, hypertension, STEMI, and Killip classification), both TyG as a categorical variable and as a continuous variable were strongly associated with the risk of MACE (*P* < 0.05). Furthermore, in model 3, which was fully adjusted for age, hypertension, STEMI, Killip classification, eGFR, aspirin, ACEI/ARB, and multivessel disease, when TyG was used as a categorical variable, the risk of MACE in the TyG-T2 and T3 groups was 1.622 times and 2.247 times higher than that in the T1 group, respectively (HR: 1.622, 95% CI: 1.169–2.251, *P* = 0.004; HR: 2.247, 95% CI: 1.639–3.082, *P* < 0.001). When TyG was treated as a continuous variable, the risk of MACE increased by 49.5% for every 1-unit increase in the TyG index (HR: 1.495, 95% CI: 1.272–1.757, *P* < 0.001).

**Table 4:** Multivariate Cox regression analysis of TyG and MACE.

Variables	Model 1			Model 2			Model 3		
	HR	95% CI	*P*-value	HR	95% CI	*P*-value	HR	95% CI	*P*-value
T1	Ref			Ref			Ref		
T2	1.535	1.109–2.124	0.010	1.661	1.198–2.303	0.002	1.622	1.169–2.251	0.004
T3	2.141	1.573–2.915	<0.001	2.304	1.680–3.160	<0.001	2.247	1.639–3.082	<0.001
TyG index	1.470	1.256–1.722	<0.001	1.505	1.282–1.767	<0.001	1.495	1.272–1.757	<0.001

Model 1: unadjusted; model 2: adjusted for age, hypertension, STEMI, and Killip classification; model 3: adjusted for age, hypertension, STEMI, Killip classification, eGFR, aspirin, ACEI/ARB, and multivessel disease.

HR, hazard ratio; CI, confidence interval; MACE, major adverse cardiovascular events; TyG, triglyceride-glucose index; T1, tertile 1; T2, tertile 2; T3, tertile 3; STEMI, ST-elevation myocardial infarction; eGFR, estimated glomerular filtration rate; ACEI, angiotensin-converting enzyme inhibitor; ARB, angiotensin II receptor blocker.

### 3.3 Hierarchical association of TyG and MACE

**Table 5** presents the hierarchical association between the TyG index and MACE. The results indicated that in the subgroup analysis, elevated TyG index levels were consistently associated with an increased risk of MACE across multiple clinical subgroups. Among patients aged <75 years, the TyG-T2 and T3 groups had significantly higher MACE risks compared to T1 (HR = 2.060, *P* = 0.014; HR = 2.865, *P* < 0.001, respectively), and similar associations were observed in those aged ≥75 years (T2: HR = 1.630, *P* = 0.019; T3: HR = 1.942, *P* = 0.001). For women, both T2 and T3 groups showed significantly elevated risks (HR = 2.347 and 2.638, both *P* ≤ 0.001), while in men, only the T3 group was significantly associated with increased MACE (HR = 2.052, *P* = 0.001). In non-STEMI patients, both the T2 and T3 groups were at significantly higher risk (HR = 1.944 and 2.244, both *P* < 0.001); among STEMI patients, the T3 group was significant (HR = 2.659, *P* < 0.001). The association remained robust in patients with hypertension (T2: HR = 1.477, *P* = 0.050; T3: HR = 1.994, *P* < 0.001) and was even stronger in those without hypertension (T2: HR = 2.308, *P* = 0.008; T3: HR = 2.899, *P* = 0.001). In Killip classification I patients, both the T2 and T3 groups were associated with higher MACE risk (HR = 1.850 and 2.842, *P* = 0.011 and <0.001), while in classification II–IV, only the T3 group showed significance (HR = 1.955, *P* = 0.002). Finally, in patients with or without multivessel disease, both the T2 and T3 tertiles were significantly linked to increased MACE risk, with the strongest association seen in the T3 group without multivessel disease (HR = 2.926, *P* < 0.001).

**Table 5:** Stratified association of TyG and MACE.

Subgroups	T1	T2		T3		
	HR (95% CI)	HR (95% CI)	*P*	HR (95% CI)	*P*	*P* for trend
Age						
<75 years	Ref	2.060 (1.160–3.660)	0.014	2.865 (1.691–4.854)	<0.001	<0.001
≥75 years	Ref	1.630 (1.083–2.453)	0.019	1.942 (1.291–2.922)	0.001	0.004
Gender						
Male	Ref	1.132 (0.709–1.807)	0.603	2.052 (1.354–3.109)	0.001	0.002
Female	Ref	2.347 (1.443–3.818)	0.001	2.638 (1.620–4.297)	<0.001	<0.001
STEMI						
Yes	Ref	1.246 (0.713–2.176)	0.440	2.659 (1.583–4.468)	<0.001	<0.001
No	Ref	1.944 (1.294–2.922)	0.001	2.244 (1.512–3.332)	<0.001	<0.001
Hypertension						
Yes	Ref	1.477 (1.000–2.184)	0.050	1.994 (1.381–2.879)	<0.001	0.001
No	Ref	2.308 (1.245–4.279)	0.008	2.899 (1.520–5.532)	0.001	0.002
Killip classification						
I	Ref	1.850 (1.154–2.964)	0.011	2.842 (1.761–4.585)	<0.001	<0.001
II–IV	Ref	1.446 (0.915–2.284)	0.114	1.955 (1.284–2.976)	0.002	0.006
Multivessel disease						
Yes	Ref	1.646 (1.043–2.596)	0.032	1.850 (1.188–2.882)	0.007	0.016
No	Ref	1.714 (1.055–2.784)	0.030	2.926 (1.884–4.546)	<0.001	<0.001

HR, hazard ratio; CI, confidence interval; MACE, major adverse cardiovascular events; TyG, triglyceride-glucose index; T1, tertile 1; T2, tertile 2; T3, tertile 3; STEMI, ST-elevation myocardial infarction.

### 3.4 ROC curves and Kaplan–Meier curve analyses

**Figure 1:**
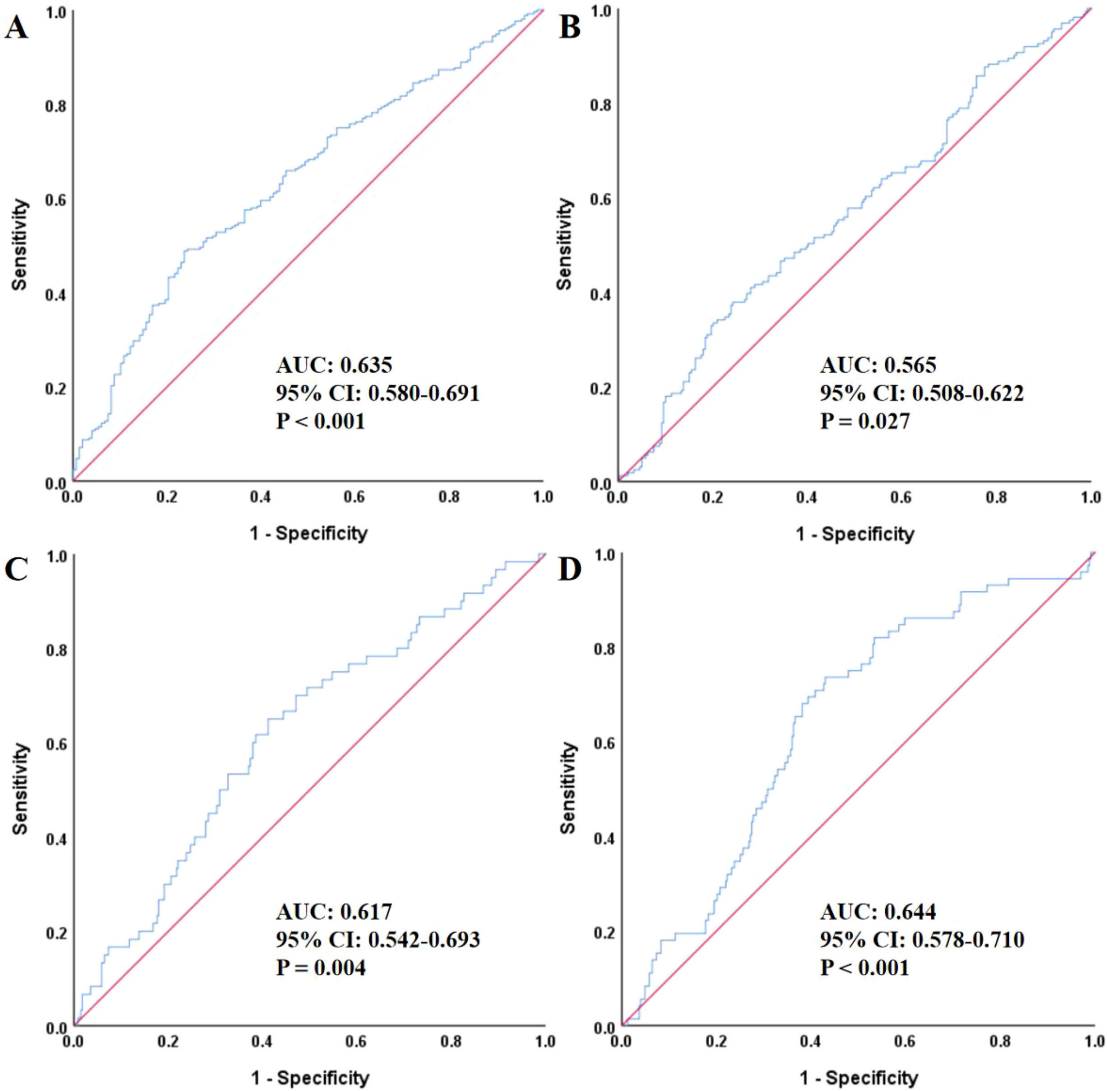
The ROC analysis of TyG for predicting MACE **(A)**, all-cause death **(B)**, non-fatal myocardial infarction **(C)**, and unplanned revascularization **(D)**. ROC, receiver operating characteristic; TyG, triglyceride-glucose index; AUC, area under the curve; CI, confidence interval; MACE, major adverse cardiovascular events.

As shown in **Figure 1**, ROC curve analysis demonstrated that the TyG index was a significant predictor for the risk of MACE (AUC: 0.635, 95% CI: 0.580–0.691, *P* < 0.001). It also predicted all-cause death (AUC: 0.565, 95% CI: 0.508–0.622, *P* = 0.027), new-onset myocardial infarction (AUC: 0.617, 95% CI: 0.542–0.693, *P* = 0.004), and second PCI (AUC: 0.644, 95% CI: 0.578–0.710, *P* < 0.001).

**Figure 2:**
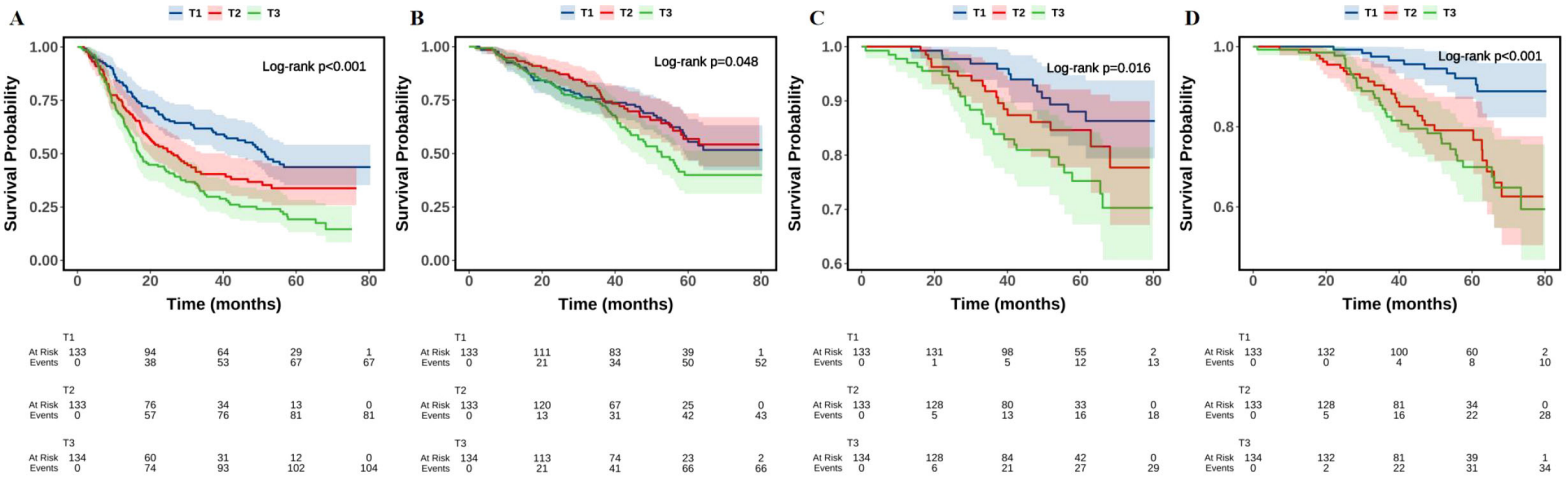
The Kaplan–Meier analysis of TyG with MACE **(A)**, all-cause death **(B)**, non-fatal myocardial infarction **(C)**, and unplanned revascularization **(D)**. TyG, triglyceride-glucose index; MACE, major adverse cardiovascular events.

Additionally, as shown in **Figure 2**, the Kaplan–Meier survival curves revealed statistically significant differences in the survival probabilities for MACE, all-cause death, non-fatal myocardial infarction, and unplanned revascularization across the three TyG index groups over time (log-rank *P* < 0.05). Notably, patients in the TyG T3 group demonstrated the steepest decline in event-free survival. The estimated HRs for MACE from Kaplan–Meier analysis were 1.535 (95% CI: 1.109–2.124, *P* = 0.010) for the TyG T2 group and 2.141 (95% CI: 1.573–2.915, *P* < 0.001) for the TyG T3 group, both compared with the T1 group.

## 4 Discussion

This study comprehensively investigated the association between the TyG index and the risk of MACE in patients with T2DM and HFpEF following AMI. Our findings revealed a clear and consistent relationship between elevated TyG levels and increased incidence of MACE. Patients in the highest TyG tertile (T3) had a more than twofold increased risk of MACE compared to those in the lowest tertile (T1), even after adjusting for multiple clinical confounders. Moreover, the risk of MACE increased by nearly 50% for each 1-unit rise in the TyG index. Subgroup analyses confirmed the robustness of this association across various clinical strata, including age, sex, hypertension status, Killip classification, and presence of multivessel disease. These findings were further supported by Kaplan–Meier survival curves and ROC analysis, where the TyG index demonstrated modest but significant predictive power for MACE and related outcomes.

Left ventricular dilation and dysfunction caused by ischemic heart disease—specifically, structural and functional remodeling of the left ventricle—can result in decreased LVEF or hemodynamic abnormalities. However, in many patients with ischemic heart disease, including those with CAD and coronary microvascular dysfunction, this dysfunction can be delayed, inhibited, or even reversed due to the widespread use of PCI. This phenomenon, referred to as HFpEF caused by either coronary large vessel obstruction or microvascular dysfunction, has become more widely recognized (29, 30). Increasingly, researchers have focused on the relationship between metabolic disorders and the development of HFpEF after myocardial infarction, especially in the context of glucose metabolism, a field that remains underexplored (31, 32).

In our study, the clinical characteristics grouped according to TyG tertiles revealed statistically significant differences in outcomes such as all-cause death, MACE, non-fatal myocardial infarction, and unplanned revascularization among the three TyG groups. The incidence of MACE, non-fatal myocardial infarction, and unplanned revascularization increased with higher TyG levels. Specifically, in patients with T2DM and HFpEF following AMI, those with a TyG index reaching or exceeding 9.51 (in the T3 group) should be closely monitored for potential MACE, non-fatal myocardial infarction, and unplanned revascularization events. After adjusting for confounding factors, the TyG index remained an independent predictor of MACE in this population.

While previous studies have not extensively investigated the correlation between the TyG index and ischemia-induced HFpEF or its adverse outcomes, multiple studies have reported correlations between the TyG index and various CVD as well as the risk of cardiovascular events. For instance, Lyu et al. (33) found a non-linear relationship between the TyG-BMI index and all-cause mortality and HF-related rehospitalizations in HF patients. They reported an inverse “J”-shaped curve, where the risk of all-cause mortality decreased when the TyG-BMI index was below 240.0. Similarly, Guo et al. (34) identified TyG and TG/HDL-C as significant predictors of in-hospital mortality in non-diabetic AMI patients. This finding aligns with the results of our study, where TyG remained a key predictor for poor outcomes in patients with T2DM following AMI. Furthermore, Wang et al. (35) demonstrated that the TyG index independently predicted future MACE in diabetic patients with acute coronary syndrome (ACS), with Kaplan–Meier survival curves showing significant event-free survival differences between TyG quartiles. In our study, the stratified analysis demonstrated that elevated TyG index levels were consistently associated with an increased risk of MACE across multiple clinical subgroups. Among patients aged <75 years, the risk of MACE in the TyG T2 and T3 groups was 2.060 and 2.865 times higher than in the T1 group, respectively. In those aged ≥75 years, the risk was 1.630 times higher in T2 and 1.942 times higher in T3 compared to T1. In terms of sex, women in the T2 and T3 groups had 2.347-fold and 2.638-fold higher risks, respectively. Among men, only the T3 group showed a significant increase in risk (2.052-fold). In patients without STEMI, the T2 and T3 groups had 1.944-fold and 2.244-fold higher risks, respectively, while in STEMI patients, the T3 group showed a 2.659-fold increase. For patients with hypertension, the MACE risk was 1.477 times higher in T2 and 1.994 times higher in T3. Among those without hypertension, the risk increased to 2.308 times in T2 and 2.899 times in T3. Among patients with Killip classification I, the T2 and T3 groups had 1.850-fold and 2.842-fold higher risks, respectively. In those with Killip classification II–IV, only the T3 group showed a notable increase (1.955-fold). For patients with or without multivessel disease, both T2 and T3 groups demonstrated elevated MACE risks. Notably, in patients without multivessel disease, the T3 group had the highest risk, with a 2.926-fold increase. In summary, the TyG index was positively associated with MACE across various subgroups, with particularly stronger predictive value in women, non-STEMI patients, those without hypertension, and those without multivessel disease—highlighting its potential utility in risk stratification for targeted management in high-risk populations.

Beyond its cardiovascular implications, the TyG index has been explored as a non-invasive marker for various diseases. Liu and colleagues found that the TyG index was an effective predictor for non-alcoholic fatty liver disease and related hepatic conditions, including hepatic fibrosis, when coupled with TyG-derived indices like TyG-BMI (36, 37). Additionally, research by Jiang et al. (38) suggested that the TyG index was causally associated with a reduced stroke risk, a finding that aligns with our results. In our study, ROC curve analysis revealed that the TyG index significantly predicted the risk of MACE, all-cause death, non-fatal myocardial infarction, and unplanned revascularization, all with statistically significant predictive value. Moreover, the Kaplan–Meier survival curves showed significant differences between the TyG tertiles in the survival probabilities for MACE, all-cause death, non-fatal myocardial infarction, and unplanned revascularization over time. Patients in the higher TyG groups exhibited the fastest decline in survival probability, suggesting that a higher TyG index (above 9.51) correlates with worse clinical prognosis. In conclusion, while the TyG index has been linked to the prediction of a range of diseases, including CVD, liver fibrosis, and stroke, its association with ischemia-induced HFpEF remains underexplored. However, our study demonstrated that the TyG index was significantly correlated with the occurrence of MACE in T2DM patients with AMI and HFpEF. Therefore, clinicians should maintain a high level of vigilance for MACE, non-fatal myocardial infarction, and unplanned revascularization in patients with higher TyG indices, particularly when the index exceeds 9.51.

The mechanisms by which the TyG index contributes to MACE in HFpEF patients following AMI are likely multifactorial. First, TyG is a recognized surrogate marker of IR, a metabolic state that promotes myocardial lipid accumulation, fibrosis, and impaired ventricular relaxation, all of which contribute to diastolic dysfunction and the development of HFpEF (39–42). Second, elevated TyG levels have been associated with microvascular dysfunction, particularly in diabetic populations. This dysfunction, characterized by reduced nitric oxide bioavailability and endothelial inflammation, leads to coronary microcirculatory impairment, exacerbating myocardial ischemia and remodeling (43, 44). Third, IR-induced alterations in myocardial calcium handling and activation of profibrotic signaling pathways promote left ventricular hypertrophy and reduced compliance, further worsening diastolic performance (45, 46). These pathophysiologic processes—IR, microvascular dysfunction, and diastolic impairment—together may explain the observed association between higher TyG index values and increased MACE risk in HFpEF patients. Our findings underscore the importance of early glycemic-lipid metabolic assessment and intervention, especially in T2DM patients post-AMI with preserved ejection fraction, to mitigate cardiovascular risk and improve long-term outcomes.

This study had several limitations. First, being retrospective in nature, selection bias may be unavoidable. Second, patients with HFpEF were primarily diagnosed using transthoracic echocardiography, which lacks the sensitivity of exercise stress echocardiography and may lead to missed diagnoses. Third, the lack of statistical significance for some survival analysis outcomes could be attributed to the small sample size and single-center design of the study. Fourth, the study population was confined to Liaoning Province, China, which may limit the generalizability of the findings to other populations. Fifth, this study did not employ propensity score matching (PSM) or inverse probability of treatment weighting (IPTW) to further control for potential confounding. The primary reasons for this were the relatively small sample size and missing data in some covariates, which limited the feasibility and stability of such analyses. While multivariable Cox regression was used to adjust for known clinical covariates, unmeasured confounding cannot be entirely excluded. Future prospective studies with larger and more diverse populations should consider incorporating PSM or IPTW to strengthen causal inference and reduce residual bias. Sixth, patients lost to follow-up and those who did not undergo interventional procedures were excluded from the analysis. While this was done to ensure data completeness and treatment consistency, it may have introduced survivorship bias, as individuals with early adverse events could have been inadvertently excluded. We acknowledge this potential bias and recommend that future studies adopt strategies such as prospective design, improved follow-up systems, or multiple imputation to minimize its impact. Seventh, although the TyG index was found to be statistically associated with MACE, its overall predictive value was limited. This suggests that, while the TyG index may have some prognostic relevance, it alone may not provide strong discriminatory power in clinical practice. Moreover, this study did not compare the TyG index with established risk scoring systems such as the Global Registry of Acute Coronary Events (GRACE) score and the Thrombolysis in Myocardial Infarction (TIMI) score, due to the unavailability of complete data required for those calculations. This lack of comparison limits the ability to contextualize the TyG index within existing clinical risk assessment frameworks. Future studies should include these established tools to better evaluate the added value of the TyG index in cardiovascular risk stratification. Eighth, while the TyG index was found to be associated with MACE, the underlying biological mechanisms—such as insulin resistance, chronic inflammation, or endothelial dysfunction—were not directly investigated in this study. As this was a retrospective analysis based on routine clinical records, mechanistic biomarkers such as fasting insulin (for Homeostasis Model Assessment of Insulin Resistance), inflammatory cytokines (e.g., interleukin-6, tumor necrosis factor-alpha), or markers of oxidative stress were not collected. This limits the ability to explore the potential pathophysiological pathways linking TyG to adverse cardiovascular outcomes. Future prospective studies incorporating metabolic and inflammatory biomarkers are warranted to better elucidate the biological basis of the observed associations. Lastly, although the TyG index shows promise in predicting and assessing various diseases, there remains no standardized range or critical value for the index across studies. Further research with larger, multicenter, and prospective designs is necessary to clarify the diagnostic cutoff points and prognostic value of the TyG index in different diseases.

## 5 Conclusions

Our study found that in T2DM patients with HFpEF combined with AMI, the incidence of MACE was higher, and the prognosis worsened as the TyG index increased. The TyG index proved to be an independent predictor of MACE and could serve as a valuable tool for risk stratification and prognosis in this population. Clinicians should be particularly alert to the risks associated with left ventricular dysfunction in patients with elevated TyG indices during the management of AMI.

## Data Availability

The original contributions presented in the study are included in the article/supplementary material. Further inquiries can be directed to the corresponding authors.

## References

[1] Lenselink C Ricken KWLM Groot HE de Bruijne TJ Hendriks T van der Harst P. Incidence and predictors of heart failure with reduced and preserved ejection fraction after ST-elevation myocardial infarction in the contemporary era of early percutaneous coronary intervention. Eur J Heart Fail. (2024) 26:1142–9. doi: 10.1002/ejhf.3225, PMID: 38576163

[2] Jenča D Melenovský V Stehlik J Staněk V Kettner J Kautzner J. Heart failure after myocardial infarction: incidence and predictors. ESC Heart Fail. (2021) 8:222–37. doi: 10.1002/ehf2.13144, PMID: 33319509 PMC7835562

[3] Salari N Morddarvanjoghi F Abdolmaleki A Rasoulpoor S Khaleghi AA Hezarkhani LA. The global prevalence of myocardial infarction: a systematic review and meta-analysis. BMC Cardiovasc Disord. (2023) 23:206. doi: 10.1186/s12872-023-03231-w, PMID: 37087452 PMC10122825

[4] Yusuf S Hawken S Ounpuu S Dans T Avezum A Lanas F. Effect of potentially modifiable risk factors associated with myocardial infarction in 52 countries (the INTERHEART study): case-control study. Lancet. (2004) 364:937–52. doi: 10.1016/S0140-6736(04)17018-9, PMID: 15364185

[5] Ban J Ma RM Liu A Wang QH Chen C Sun Q. Ambient PM2.5 and acute incidence of myocardial infarction in China: a case-crossover study and health impact assessment. Cardiol Plus. (2023) 8:111–7. doi: 10.1097/CP9.0000000000000047

[6] Yang CD Shen Y Ding FH Yang ZK Hu J Shen WF. Visit-to-visit fasting plasma glucose variability is associated with left ventricular adverse remodeling in diabetic patients with STEMI. Cardiovasc Diabetol. (2020) 19:131. doi: 10.1186/s12933-020-01112-6, PMID: 32878604 PMC7469406

[7] Tomasik A Nabrdalik K Kwiendacz H Radzik E Pigoń K Młyńczak T. Effect of diabetes mellitus and left ventricular perfusion on frequency of development of heart failure and/or all-cause mortality late after acute myocardial infarction. Am J Cardiol. (2021) 140:25–32. doi: 10.1016/j.amjcard.2020.10.051, PMID: 33144164

[8] Bouisset F Bataille V Schiele F Puymirat E Fayol A Simon T. Type 2 diabetes mellitus in acute myocardial infarction: a persistent significant burden on long-term mortality. Front Cardiovasc Med. (2024) 11:1401569. doi: 10.3389/fcvm.2024.1401569, PMID: 38932992 PMC11204119

[9] Liu JX Zhou L Wang XS Dong JZ. Sex differences in quality of life and clinical outcomes in patients with heart failure. Cardiovasc Innov Appl. (2023) 8:1–10. doi: 10.15212/CVIA.2023.0046

[10] Heidenreich PA Bozkurt B Aguilar D Allen LA Byun JJ Colvin MM. 2022 AHA/ACC/HFSA guideline for the management of heart failure: A report of the american college of cardiology/american heart association joint committee on clinical practice guidelines. Circulation. (2022) 145:e895–e1032. doi: 10.1161/CIR.0000000000001063, PMID: 35363499

[11] Vilaro JR. Heart failure with preserved ejection fraction: important things to know about the stiff heart. Cardiovasc Innov Appl. (2023) 8:1–4. doi: 10.15212/CVIA.2023.0058

[12] Kosmas CE Bousvarou MD Kostara CE Papakonstantinou EJ Salamou E Guzman E. Insulin resistance and cardiovascular disease. J Int Med Res. (2023) 51:3000605231164548. doi: 10.1177/03000605231164548, PMID: 36994866 PMC10069006

[13] Kurniawan LB. Triglyceride-glucose index as A biomarker of insulin resistance, diabetes mellitus, metabolic syndrome, and cardiovascular disease: A review. EJIFCC. (2024) 35:44–51., PMID: 38706737 PMC11063788

[14] Wang Y Yang W Jiang X. Association between triglyceride-glucose index and hypertension: A meta-analysis. Front Cardiovasc Med. (2021) 8:644035. doi: 10.3389/fcvm.2021.644035, PMID: 34136539 PMC8200397

[15] Nilofer Sagana MK Arul Senghor KA Vinodhini VM P R. Irisin and triglyceride glucose index as markers of dyslipidemia in young adults. Indian J Clin Biochem. (2024) 39:136–41. doi: 10.1007/s12291-022-01083-3, PMID: 38223008 PMC10784433

[16] Yang S Wang Z. The triglyceride-glucose index is a promising predictor for the risk of cardiovascular disease in the diabetic population aged ≥ge years in the United States: a retrospective cohort study from NHANES (2007-2016). Front Endocrinol (Lausanne). (2025) 16:1475590. doi: 10.3389/fendo.2025.1475590, PMID: 40060380 PMC11885141

[17] Lopez-Jaramillo P Gomez-Arbelaez D Martinez-Bello D Abat MEM Alhabib KF Avezum Á. Association of the triglyceride glucose index as a measure of insulin resistance with mortality and cardiovascular disease in populations from five continents (PURE study): a prospective cohort study. Lancet Healthy Longev. (2023) 4:e23–33. doi: 10.1016/S2666-7568(22)00247-1, PMID: 36521498

[18] Sun T Huang X Zhang B Ma M Chen Z Zhao Z. Prognostic significance of the triglyceride-glucose index for patients with ischemic heart failure after percutaneous coronary intervention. Front Endocrinol (Lausanne). (2023) 14:1100399. doi: 10.3389/fendo.2023.1100399, PMID: 36814584 PMC9939475

[19] Ruan H Duan S He L Wang Y Yao Z Pan L. The Incremental Prognostic Value of Incorporating the Triglyceride-Glucose Index into the Traditional Cardiovascular Risk Factors for the Long-term Prognosis in Ischemic Cardiomyopathy Patients with HFpEF following Coronary Artery Bypass Grafting: A Multicenter Cohort Study. J Atheroscler Thromb. (2025). doi: 10.5551/jat.65654, PMID: 40222904 PMC12504032

[20] McEvoy JW McCarthy CP Bruno RM Brouwers S Canavan MD Ceconi C. 2024 ESC Guidelines for the management of elevated blood pressure and hypertension. Eur Heart J. (2024) 45:3912–4018. doi: 10.1093/eurheartj/ehae178, PMID: 39210715

[21] American Diabetes Association Professional Practice Committee. 2. Diagnosis and classification of diabetes: standards of care in diabetes-2025. Diabetes Care. (2025) 48:S27–49. doi: 10.2337/dc25-S002, PMID: 39651986 PMC11635041

[22] Kleindorfer DO Towfighi A Chaturvedi S Cockroft KM Gutierrez J Lombardi-Hill D. 2021 guideline for the prevention of stroke in patients with stroke and transient ischemic attack: A guideline from the american heart association/american stroke association. Stroke. (2021) 52:e364–467. doi: 10.1161/STR.0000000000000375, PMID: 34024117

[23] Joglar JA Chung MK Armbruster AL Benjamin EJ Chyou JY Cronin EM. 2023 ACC/AHA/ACCP/HRS guideline for the diagnosis and management of atrial fibrillation: A report of the american college of cardiology/american heart association joint committee on clinical practice guidelines. Circulation. (2024) 149:e1–e156. doi: 10.1161/CIR.0000000000001193, PMID: 38033089 PMC11095842

[24] McDonagh TA Metra M Adamo M Gardner RS Baumbach A Böhm M. 2021 ESC Guidelines for the diagnosis and treatment of acute and chronic heart failure. Eur Heart J. (2021) 42:3599–726. doi: 10.1093/eurheartj/ehab368, PMID: 34447992

[25] Rao SV O'Donoghue ML Ruel M Rab T Tamis-Holland JE Alexander JH. 2025 ACC/AHA/ACEP/NAEMSP/SCAI guideline for the management of patients with acute coronary syndromes: A report of the american college of cardiology/american heart association joint committee on clinical practice guidelines. Circulation. (2025) 151:e771–862. doi: 10.1161/CIR.0000000000001309, PMID: 40014670

[26] Itzahki Ben Zadok O Ben-Gal T Abelow A Shechter A Zusman O Iakobishvili Z. Temporal trends in the characteristics, management and outcomes of patients with acute coronary syndrome according to their killip class. Am J Cardiol. (2019) 124:1862–8. doi: 10.1016/j.amjcard.2019.09.012, PMID: 31685211

[27] Ma YC Zuo L Chen JH Luo Q Yu XQ Li Y. Modified glomerular filtration rate estimating equation for Chinese patients with chronic kidney disease. J Am Soc Nephrol. (2006) 17:2937–44. doi: 10.1681/ASN.2006040368, PMID: 16988059

[28] Simental-Mendía LE Rodríguez-Morán M Guerrero-Romero F. The product of fasting glucose and triglycerides as surrogate for identifying insulin resistance in apparently healthy subjects. Metab Syndr Relat Disord. (2008) 6:299–304. doi: 10.1089/met.2008.0034, PMID: 19067533

[29] Sinha A Rahman H Perera D. Coronary microvascular dysfunction and heart failure with preserved ejection fraction: what are the mechanistic links? Curr Opin Cardiol. (2023) 38:521–6. doi: 10.1097/HCO.0000000000001082, PMID: 37668191 PMC10552827

[30] Velollari O Rommel KP Kresoja KP Lurz P Gori T. Focusing on microvascular function in heart failure with preserved ejection fraction. Heart Fail Rev. (2025) 30:493–503. doi: 10.1007/s10741-024-10479-7, PMID: 39804445 PMC11992002

[31] Luo L Zuo Y Dai L. Metabolic rewiring and inter-organ crosstalk in diabetic HFpEF. Cardiovasc Diabetol. (2025) 24:155. doi: 10.1186/s12933-025-02707-7, PMID: 40186193 PMC11971867

[32] Hahn VS Petucci C Kim MS Bedi KC Jr Wang H Mishra S. Myocardial metabolomics of human heart failure with preserved ejection fraction. Circulation. (2023) 147:1147–61. doi: 10.1161/CIRCULATIONAHA.122.061846, PMID: 36856044 PMC11059242

[33] Lyu L Wang X Xu J Liu Z He Y Zhu W. Association between triglyceride glucose-body mass index and long-term adverse outcomes of heart failure patients with coronary heart disease. Cardiovasc Diabetol. (2024) 23:162. doi: 10.1186/s12933-024-02213-2, PMID: 38724999 PMC11080126

[34] Guo J Ji Z Carvalho A Qian L Ji J Jiang Y. The triglycerides-glucose index and the triglycerides to high-density lipoprotein cholesterol ratio are both effective predictors of in-hospital death in non-diabetic patients with AMI. PeerJ. (2022) 10:e14346. doi: 10.7717/peerj.14346, PMID: 36438585 PMC9686411

[35] Wang L Cong HL Zhang JX Hu YC Wei A Zhang YY. Triglyceride-glucose index predicts adverse cardiovascular events in patients with diabetes and acute coronary syndrome. Cardiovasc Diabetol. (2020) 19:80. doi: 10.1186/s12933-020-01054-z, PMID: 32534586 PMC7293784

[36] Liu H Chen J Qin Q Yan S Wang Y Li J. Association between TyG index trajectory and new-onset lean NAFLD: a longitudinal study. Front Endocrinol (Lausanne). (2024) 15:1321922. doi: 10.3389/fendo.2024.1321922, PMID: 38476672 PMC10927994

[37] Xue Y Xu J Li M Gao Y. Potential screening indicators for early diagnosis of NAFLD/MAFLD and liver fibrosis: Triglyceride glucose index-related parameters. Front Endocrinol (Lausanne). (2022) 13:951689. doi: 10.3389/fendo.2022.951689, PMID: 36120429 PMC9478620

[38] Jiang Y Shen J Chen P Cai J Zhao Y Liang J. Association of triglyceride glucose index with stroke: from two large cohort studies and Mendelian randomization analysis. Int J Surg. (2024) 110:5409–16. doi: 10.1097/JS9.0000000000001795, PMID: 38896856 PMC11392123

[39] Nakamura M Sadoshima J. Cardiomyopathy in obesity, insulin resistance and diabetes. J Physiol. (2020) 598:2977–93. doi: 10.1113/JP276747, PMID: 30869158

[40] Caturano A Galiero R Vetrano E Sardu C Rinaldi L Russo V. Insulin-heart axis: bridging physiology to insulin resistance. Int J Mol Sci. (2024) 25:8369. doi: 10.3390/ijms25158369, PMID: 39125938 PMC11313400

[41] Dahiya R Shultz SP Dahiya A Fu J Flatley C Duncan D. Relation of reduced preclinical left ventricular diastolic function and cardiac remodeling in overweight youth to insulin resistance and inflammation. Am J Cardiol. (2015) 115:1222–8. doi: 10.1016/j.amjcard.2015.02.005, PMID: 25765589

[42] Fazio S Mercurio V Fazio V Ruvolo A Affuso F. Insulin resistance/hyperinsulinemia, neglected risk factor for the development and worsening of heart failure with preserved ejection fraction. Biomedicines. (2024) 12:806. doi: 10.3390/biomedicines12040806, PMID: 38672161 PMC11047865

[43] Takei Y Tomiyama H Tanaka N Yamashina A Chikamori T. Association between insulin resistance, oxidative stress, sympathetic activity and coronary microvascular function in patients with early stage impaired glucose metabolism. Circ J. (2022) 86:866–73. doi: 10.1253/circj.CJ-21-0549, PMID: 34789613

[44] Zhou D Lin S Liu Z Yuan J Ren H Tan H. Metabolic syndrome, left ventricular diastolic dysfunction and heart failure with preserved ejective fraction. Front Endocrinol (Lausanne). (2025) 16:1544908. doi: 10.3389/fendo.2025.1544908, PMID: 40297180 PMC12034560

[45] Miranda-Silva D Wüst RCI Conceição G Gonçalves-Rodrigues P Gonçalves N Gonçalves A. Disturbed cardiac mitochondrial and cytosolic calcium handling in a metabolic risk-related rat model of heart failure with preserved ejection fraction. Acta Physiol (Oxf). (2020) 228:e13378. doi: 10.1111/apha.13378, PMID: 31520455 PMC7064935

[46] Carvajal K Balderas-Villalobos J Bello-Sanchez MD Phillips-Farfán B Molina-Muñoz T Aldana-Quintero H. Ca(2+) mishandling and cardiac dysfunction in obesity and insulin resistance: role of oxidative stress. Cell Calcium. (2014) 56:408–15. doi: 10.1016/j.ceca.2014.08.003, PMID: 25168907

